# Effect of health education on mood and pregnancy rate among infertile patients undergoing assisted reproduction: A systematic review and meta-analysis

**DOI:** 10.1097/MD.0000000000046165

**Published:** 2025-11-21

**Authors:** Ruihua Xie, Fangfang Xie

**Affiliations:** a Chengdu Women’s and Children’s Central Hospital, School of Medicine, University of Electronic Science and Technology of China, Chengdu, China; b Nursing Department, Sichuan Jinxin Xinan Women and Children Hospital, Chengdu, China.

**Keywords:** assisted reproduction, embryo transfer, health cognition, infertility

## Abstract

**Background:**

Infertility is associated with significant psychological distress, and health education is widely used to improve reproductive knowledge and support treatment outcomes in patients undergoing assisted reproductive technology (ART). However, evidence regarding its effectiveness in enhancing psychological well-being and pregnancy outcomes remains limited. This systematic review and meta-analysis aimed to evaluate the impact of health education on cognitive outcomes, psychological state, and pregnancy rates in infertile patients receiving ART.

**Methods:**

A systematic literature search was conducted in the PubMed and China National Knowledge Infrastructure databases from inception to October 2023, using combinations of terms such as “ART,” “Embryo Transfer,” “Reproductive Techniques, Assisted,” “In vitro fertilization and embryo transfer,” “Randomized controlled trial,” and “Infertility.” Eligible studies were randomized controlled trials that investigated health education interventions in ART patients. Meta-analyses were performed using Review Manager version 5.3.

**Results:**

A total of eleven randomized controlled trials met the inclusion criteria, encompassing 1324 participants. Seven studies reported health outcome scores, showing a significant improvement following health education interventions (Z = 19.17, *P* <.001). The pooled analysis of health knowledge awareness across 4 treatment stages also yielded a Z score of 9.40 (*P* <.001). Additionally, patient satisfaction was significantly higher in the observation group (Z = 6.36, *P* <.001).

**Conclusion:**

Health education appears to significantly improve cognitive understanding, health awareness, and patient satisfaction among individuals undergoing ART. These findings support the incorporation of structured health education at multiple stages of ART to enhance psychological adaptation and clinical outcomes.

## 1. Introduction

Infertility is defined as the failure to conceive after 1 year of regular, unprotected sexual intercourse,^[1]^ and its incidence increases annually. The etiology of infertility is multifactorial, with psychological stress related to the desire to conceive recognized as a contributing factor.^[2]^ With the development of bio-psycho-social medicine, more attention has been directed toward the influence of psychological factors on infertility, and the relationship between infertility and psychological factors has been widely explored.^[3]^ Many couples face a prolonged history of infertility prior to initiating assisted reproductive technology (ART), often after multiple unsuccessful treatment attempts.^[4]^ These experiences impose a significant psychological burden, as patients frequently perceive ART as a last resort with no viable alternatives.^[5]^ In addition, ART outcomes are influenced by a range of biological and external factors, and many patients require repeated cycles of treatment while experiencing various overlapping symptoms.^[6]^ Under persistent psychological distress of chronic anxiety and depression, patients may exhibit emotional responses such as fear, panic, denial, pleasure, warmth, feelings of loss, and paranoia, all of which can influence infertility.^[7]^ While psychological factors are important, infertility remains a complex condition affected by numerous variables. As the prevalence of infertility continues to rise, the psychological challenges associated with both the condition and its treatment have garnered substantial attention, prompting efforts to improve the mental well-being of affected individuals.^[8]^ Health education is one of the most widely used interventions to enhance reproductive health literacy, reshape patients’ understanding of infertility, and promote positive treatment outcomes.^[9]^ In the context of ART, health education plays an important role in alleviating clinical symptoms, reducing inflammatory indicators, and achieving better treatment responses with lower disease activity.

According to a recent report by the World Health Organization, the global incidence of infertility is increasing rapidly, with approximately 17.5% of the adult population, equivalent to 1 in 6 individuals, affected by infertility. In China, the prevalence is even higher, with an estimated 25% of couples experiencing infertility.^[10]^ The treatment process for infertility is often prolonged and complex, and the uncertainty of pregnancy outcome causes anxiety and depression, which affects the quality of life, reduces treatment adherence, and ultimately compromises treatment efficacy.^[11]^ During ART, patients often undergo pressure from society and family, and experience anxiety and depression regarding the outcome.^[12]^ Some studies have pointed out that such psychological distress can adversely affect ART success rates.^[13]^ World Health Organization data also show that infertility affects approximately 20% of couples of reproductive age, making it the third most prevalent disease globally.^[14]^ ART remains the most common therapeutic approach, enabling many individuals to achieve parenthood.^[15]^ Infertility predominantly affects younger populations and arises from a combination of psychological stress, physical fitness, and external factors.^[16]^ In response to the introduction of China’s 2-child and 3-child policies, a large number of older women have expressed a strong desire to conceive, leading to a continuous increase in ART utilization each year.^[17]^ This trend not only influences individual and family trajectories but also contributes to improved fertility rates, optimization of population structure, and broader impacts on China’s demographic and socio-economic development^[18]^ However, many ART patients continue to face substantial psychological stress from family, society, and treatment-related challenges, resulting in negative emotional states, psychological disorders, and difficulties with social adjustment.^[19–21]^

Health education is a structured process through which nursing professionals are equipped to support patients, families, or communities in maintaining or restoring optimal health. During infertility treatment, patients experience physiological and psychological stress reactions, resulting in a wide range of consultation needs.^[22]^ Health education during this period focuses on patient-centered communication and active engagement, often facilitated through online interactive platforms. The progressive transmission of tailored professional knowledge, combined with ongoing supervision and feedback, addresses the diverse informational needs of patients from different cultural backgrounds. This approach enhances treatment adherence, strengthens coping mechanisms, and helps alleviate the psychological burden associated with infertility.^[23–28]^ Furthermore, convenient feedback channels improve the communication between patients and healthcare providers. Patients can select from multiple modes of interaction to reach medical staff promptly, enabling timely responses to their concerns and effectively reducing anxiety.^[29]^

Health education represents an evolving model of care that emphasizes personalized guidance based on patients’ individual needs or disease characteristics.^[30]^ Its implementation promotes greater standardization and scientific rigor in the delivery of educational content, allowing patients to gain a more comprehensive understanding of ART.^[31]^ By integrating structured health education into clinical workflows, omission caused by the heavy workload of nursing staff can be minimized, thereby enhancing the overall quality and consistency of patient education.^[32]^ In addition, the diversification of health education formats, particularly through the integration of information technology, facilitates improved patient comprehension, corrects misconceptions, and strengthens knowledge related to ART.^[33]^ During the initial face-to-face ART treatment, some patients may lose confidence due to limited understanding of the assisted conception process and uncertain results. This fear of failure may lead to negative emotions affecting the treatment results.^[34]^ Health education, when aligned with defined treatment phases, clarifies key care points at each stage, aids patients in navigating the complex treatment process, and enhances their confidence in its success.^[35,36]^ It is hypothesized that patients who receive structured health education are more likely to experience improved emotional well-being and higher pregnancy rates following ART. Furthermore, psychological interventions for women with infertility have been shown to significantly improve pregnancy outcomes while reducing levels of anxiety and depression.^[37]^ Counseling has also been associated with increased pregnancy rates and is considered a promising therapeutic option, particularly for individuals who are not undergoing medical treatment for infertility.^[38]^

Currently, there is a lack of empirical evidence supporting the hypothesis that health education and supportive care services improve psychological well-being and pregnancy outcomes in infertile patients undergoing ART. This study aimed to address this gap by conducting a meta-analysis to evaluate the effects of health education on reducing negative emotions and increasing the likelihood of successful pregnancy in this population. Specifically, we systematically examined the effects of health education on psychological distress and pregnancy rates among infertile patients receiving ART. The primary objective was to provide evidence-based insights through meta-analytic methods to inform and support clinical decision-making in the management of infertility.

## 2. Materials and methods

### 2.1. Literature search

A comprehensive search was conducted across multiple databases, including PubMed, Embase, Web of Science, CNT, Wanfang, and the Chinese scientific and technology journal database, covering literature from database inception to October 31, 2023. The included studies were randomized controlled trials (RCTs) investigating the effects of health education in patients undergoing ART. The search strategy combined subject terms and free-text terms, using combinations such as “ART, Embryo Transfer, Reproductive Techniques, Assisted,” “In vitro fertilization and embryo transfer,” “ Randomized controlled trial,” “health education,” “assisted reproduction,” “infertility,” “embryo transfer,” “and” or “or.” This study is a systematic review and meta-analysis of previously published literature, designed according to the PRISMA guidelines, as it did not involve the collection of new data from human participants. Institutional review board approval and informed consent were not required.

### 2.2. Literature inclusion and exclusion criteria

The inclusion criteria were as follows: patients diagnosed with infertility who underwent ART; randomized controlled trials published in either domestic or international databases; patients without surgical contraindications and with stable preoperative vital signs, including blood pressure, respiration, and heart rate; and studies in which the primary intervention was health education related to ART, covering cognitive improvement across 4 stages, namely pre-pregnancy preparation, embryo transfer preparation, the embryo transfer procedure itself, and post-transfer care. Studies were excluded if the full text or key outcome data could not be retrieved.

### 2.3. Quality assessment

The quality of literature included in the meta-analysis was evaluated using the Cochrane collaborator, which incorporated selective bias, implementation bias, measurement bias, follow-up bias, random bias, blindness of outcome assessors, completeness of data, completeness of selective reporting and other potential sources of bias by 2 primary reviewers, whereby each domain was rated as “low risk,” “unclear risk,” or “high risk.” In cases of disagreement between the 2 reviewers, a third researcher was consulted to reach a consensus.

### 2.4. Literature information and data extraction

All retrieved documents were manually screened in accordance with the predefined inclusion and exclusion criteria. For each eligible study, the following information was extracted: year of publication, first author, sample size, trial design, and outcome measures. Duplicate records were identified and removed using EndNote X9 reference management software (Clarivate Analytics, Philadelphia). Two researchers independently and concurrently performed the screening and data extraction procedures. Any disagreements were resolved through discussion, and, when necessary, by consultation with a third researcher.

### 2.5. Outcomes and assessment

The primary outcomes were health cognition score, health knowledge awareness rate, and patient satisfaction. Health cognition score was defined as the quantitative assessment of patients’ understanding of ART procedures, precautions, and related health information, measured by validated or study-specific questionnaires. Health knowledge awareness rate refers to the proportion of patients providing correct responses to questions related to ART procedures and care requirements, expressed as a percentage. Patient satisfaction denoted participants’ reported satisfaction with nursing care or health education, assessed using study-specific surveys. All outcomes were collected for 4 predefined treatment stages: pre-pregnancy preparation, embryo transfer preparation, embryo transfer procedure, and post-transfer care. For each study, all results compatible with these definitions were extracted as reported, without restriction by measurement tool or time point, provided they corresponded to the relevant stage and outcome domain.

### 2.6. Statistical analysis

A pooled analysis was conducted using Review Manager 5.3 software. Heterogeneity among the included studies was assessed using the I² statistic. An *I*^2^ <50 and *P* >.05 indicated homogeneity, and warranting the use of a fixed-effect model (FEM) for estimating the combined effect size. Conversely, an *I*^2^ >50 and *P* <.05 indicated heterogeneity; and the meta-analysis was performed using a random-effects model (REM). For dichotomous variables, such as health knowledge awareness, the effect size was expressed as the odds ratio (OR) with a 95% confidence interval (CI). For continuous variables, the weighted mean difference was used as the effect size when measurement units were consistent; otherwise, the standardized mean difference (SMD) was employed, both reported with 95% CIs. The statistical significance of the pooled estimates was evaluated using the Z test. The probability *P*-value was calculated based on the u value, and α = 0.05 was used as the test level. If *P* <.05, the combined statistic was considered statistically significant. For dichotomous variables, a 95% CI that did not contain 1 (i.e., 95% CI >1 or <1) was considered statistically significant, equivalent to *P* <.05. For continuous variables, statistical significance was indicated if the 95% CI did not include 0 (i.e., 95% CI >0 or <0), also corresponding to *P* <.05.

## 3. Results

### 3.1. Literature search results and basic information

**Figure 1.**
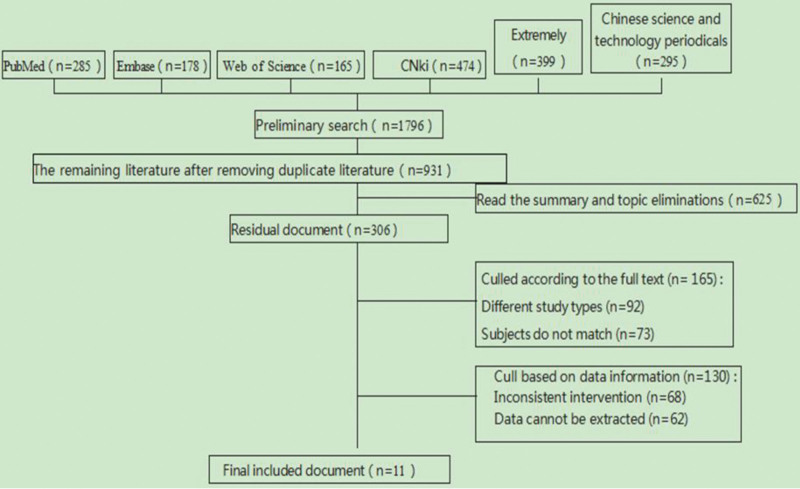
Flow chart of literature screening related to the implementation of health education for pregnant patients with assisted reproduction.

Using both Chinese and English search terms, 1796 articles were retrieved, including 285 from PubMed, 178 from Embase, 165 from Web of Science, 474 from China National Knowledge Infrastructure, and 399 from Wanfang. After the preliminary search, 865 duplicate records were removed, yielding 931 unique articles. A total of 625 articles that did not meet the inclusion criteria were excluded after reviewing the titles and abstracts. An additional 295 articles were excluded following a full-text review due to noncompliance with the inclusion criteria. Ultimately, 11 articles^[8,20,25,39–46]^ were included in the meta-analysis. The literature search results and screening process are shown in Figure 1. For each of the 11 included studies, relevant information was extracted, including author, year of publication, sample size, and patient characteristics, such as age and duration of infertility. The total sample size across all studies was 1324, including 662 participants in the control and observation groups (Table 1).

**Table 1:** Includes the literature information related to the implementation of health education in assisted reproductive patients who meet the criteria.

Author	Publication year	Intervention study		Sample size/case			
		Control group	Observation group	Control group	Observation group		
Gao Tingting^[8]^	2019	Regular education	Stage-type health education	53	53		
Wenna Chen^[39]^	2018	Routine education-care model	Stage-wise and standardized health education model	50	50		
Cheng Yun^[40]^	2018	usual care	A combined phased health education model for routine care	49	49		
Qi Ying^[41]^	2021	Routine education model	Stage-type health education	42	42		
Xu Li^[42]^	2019	Routine nursing care intervention	Stage-type health education	65	65		
Zhao Xuehan^[43]^	2020	usual care	Stage-type health education	46	46		
Roni^[44]^	2022	usual care	Joint phased health education	185	185		
Liao^[20]^	2017	Routine education model	Stage-type health education	28	28		
Sun^[45]^	2019	Regular education	Stage-type health education	53	53		
Jin Li^[46]^	2022	General education model	Stage-type health education	40	40		
Rekin’e^[25]^	2019	Regular education and nursing	Stage-type health education and nursing care	51	51		
Gao Tingting^[8]^	2019	34.3 ± 4.2	35.5 ± 4.1	–		–	Health cognitive score and satisfaction
Wenna Chen^[39]^	2018	35.2 ± 3.5	35.7 ± 3.9	3–4		2–3	Health knowledge awareness rate and satisfaction rate
Cheng Yun^[40]^	2018	34.51 ± 3.17	34.42 ± 3.06	–		–	Health knowledge awareness rate
Qi Ying^[41]^	2021	32.04 ± 3.26	31.97 ± 3.33	4.88 ± 1.38		4.81 ± 1.21	Health cognitive score and satisfaction
Xu Li^[42]^	2019	32.0 ± 4.5	31.8 ± 4.2	4.8 ± 1.2		4.7 ± 0.32	degree of satisfaction
Zhao Xuehan^[43]^	2020	33.5 ± 2.5	33.3 ± 2.8	3.6 ± 1.1		3.3 ± 1.4	Health knowledge awareness rate
Roni^[47]^	2022	31.22 ± 1.68	31.42 ± 1.74	4.23 ± 0.65		4.18 ± 0.69	Health Cognitive Score
Liao^[44]^	2017	26.17 ± 1.05	25.15 ± 1.02	–		–	Health cognitive score and satisfaction
Sun^[45]^	2019	39.47 ± 4.06	37.94 ± 4.13	–		–	Health Cognitive Score
Jin Li^[46]^	2022	36.9 ± 2.7	37.2 ± 2.9	2–5		1–4	Health cognitive score and satisfaction
Rekin’e^[25]^	2019	34.50 ± 3.16	34.41 ± 3.05	–		–	Health cognitive score and satisfaction

### 3.2. Quality evaluation of included literature

The risk-of-bias was evaluated using the Cochrane Handbook 5.0.2 risk-of-bias assessment tool, and the results were entered into Review Manager 5.3 to generate a risk of bias summary.

**Figure 2.**
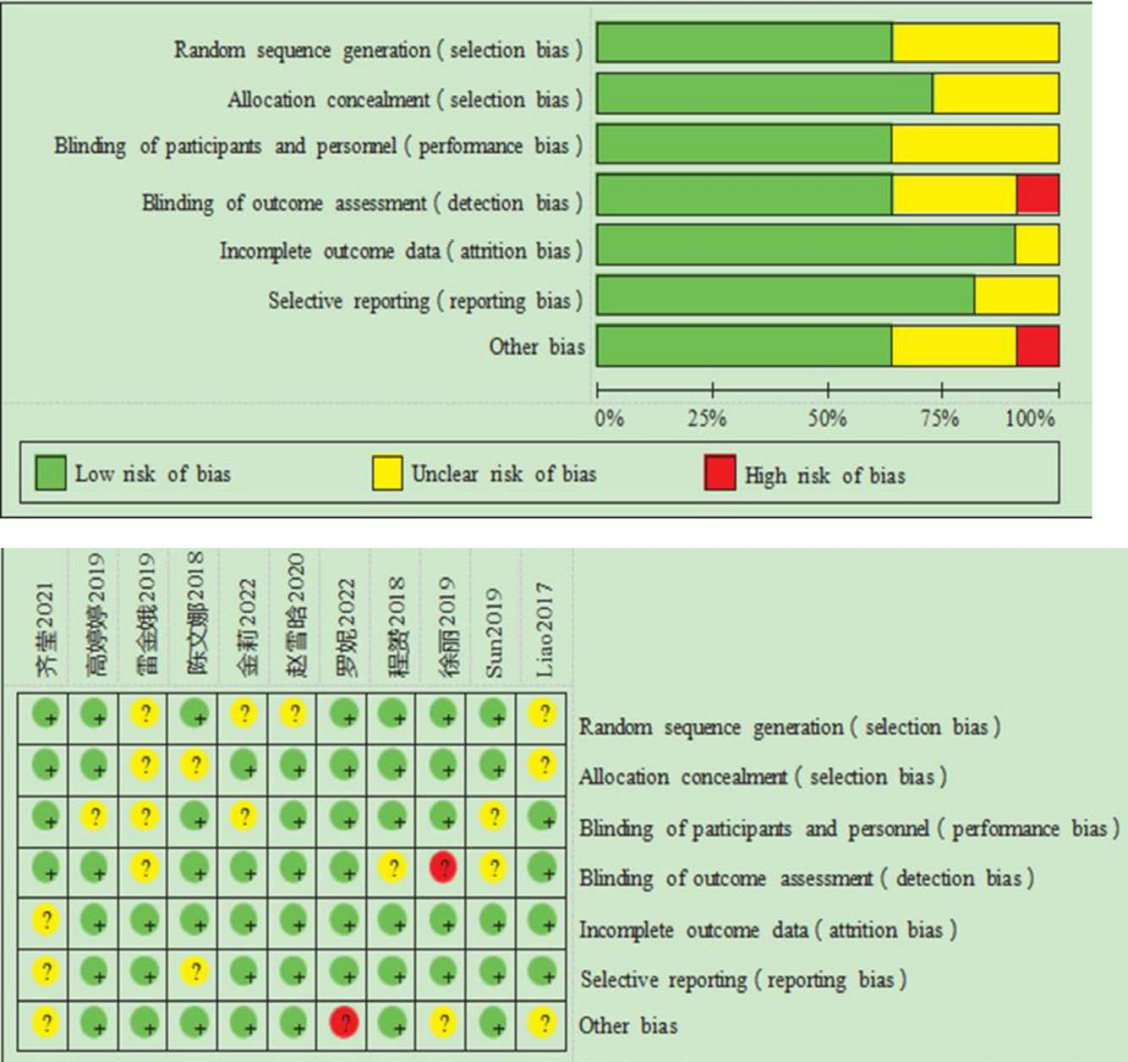
Results of risk bias evaluation corresponding to the 9 articles of the included studies. Note: The symbol “+” in the figure represents low risk bias; “?” represents unknown risk bias; “-” represents high risk bias.

1. Random sequence generation: All 11 included studies were randomized trials; however, 4 studies did not report the specific methods of randomization, resulting in an assessment of unclear risk.
2. Allocation concealment: Only 3 articles did not mention allocation concealment, indicating unclear risk.
3. Participant anonymization: Four articles did not specify whether participant blinding or anonymization was implemented, also representing an unclear risk.
4. Blinding of outcome assessors: Three articles did not report whether the outcome assessors were anonymized, indicating unclear risk, while 1 article explicitly stated that blinding was not implemented, indicating high risk. The overall risk of bias associated with the 11 included articles is illustrated in Figure 2. The included studies generally demonstrated a low risk of bias, indicating that the literature was of high methodological quality.

### 3.3. Health cognitive score

**Figure 3.**
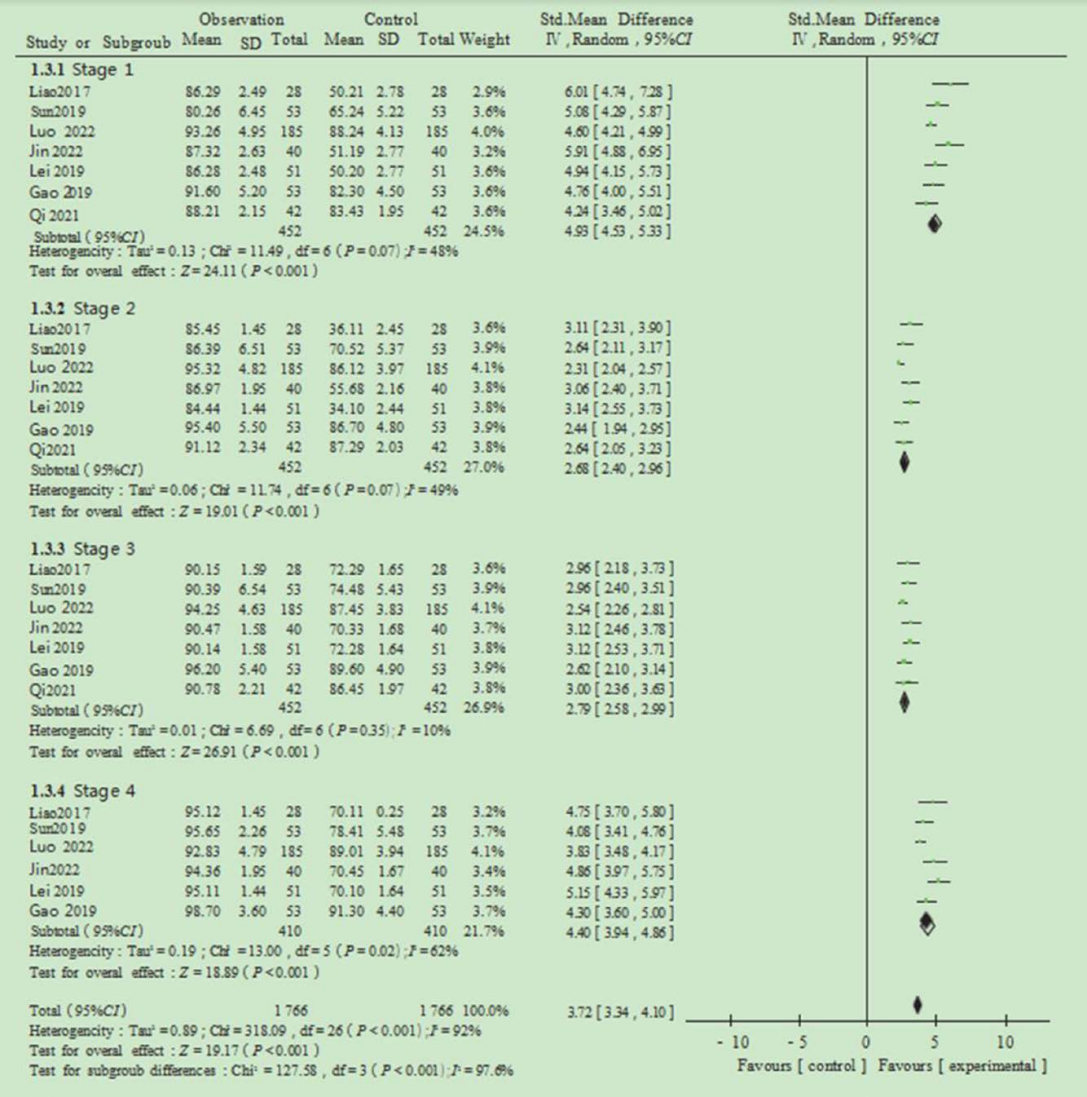
Forest plot of the effect of implementing health education on health cognitive scores of assisted reproduction patients.

Patients were analyzed across 4 stages: pre-pregnancy preparation, embryo transfer preparation, the embryo transfer procedure, and post-transfer care. Among the 11 included studies, 7 provided outcome measures for health cognition scores. In stage 1 (pre-pregnancy preparation), data from 452 patients across the control and observation groups were pooled. For the different questionnaires, SMD was used as the effect size, and the combined SMD was 4.93, with a 95% CI of 4.53 to 5.33. The heterogeneity test yielded *I*^2^ = 48% and *P* = .07, suggesting no significant heterogeneity. The REM analysis revealed a Z score of 24.11 (*P* <.001), indicating statistical significance. In stage 2 (embryo transfer preparation), both the control and observation groups included 452 patients, with a combined SMD of 2.68 and a 95% CI of 2.40 to 2.96. The heterogeneity test showed *I*^2^ = 49% and *P* = .07, again suggesting no significant heterogeneity. The REM analysis yielded a Z score of 19.01 (*P* <.001), indicating statistical significance. In stage 3 (embryo transfer procedure), both groups included 452 patients, with a combined SMD of 2.79 and a 95% CI of 2.58 to 2.99. The heterogeneity test showed *I*^2^ = 10% and *P* = .35, suggesting no significant heterogeneity. The REM analysis revealed a Z score of 26.91 (*P* <.001), indicating statistical significance. In stage 4 (post-transfer care), the combined SMD was 4.40, with a 95% CI of 3.94 to 4.86. The heterogeneity test showed *I*^2^ = 62% and *P* = .02, suggesting heterogeneity. The REM analysis yielded a Z score of 18.89 (*P* <.001), demonstrating statistical significance. The overall pooled analysis across the 4 stages resulted in a Z score of 19.17 (*P* <.001), indicating a statistically significant difference in health cognition scores between the control and observation groups (Fig. 3).

### 3.4. Rate of health knowledge awareness

**Figure 4.**
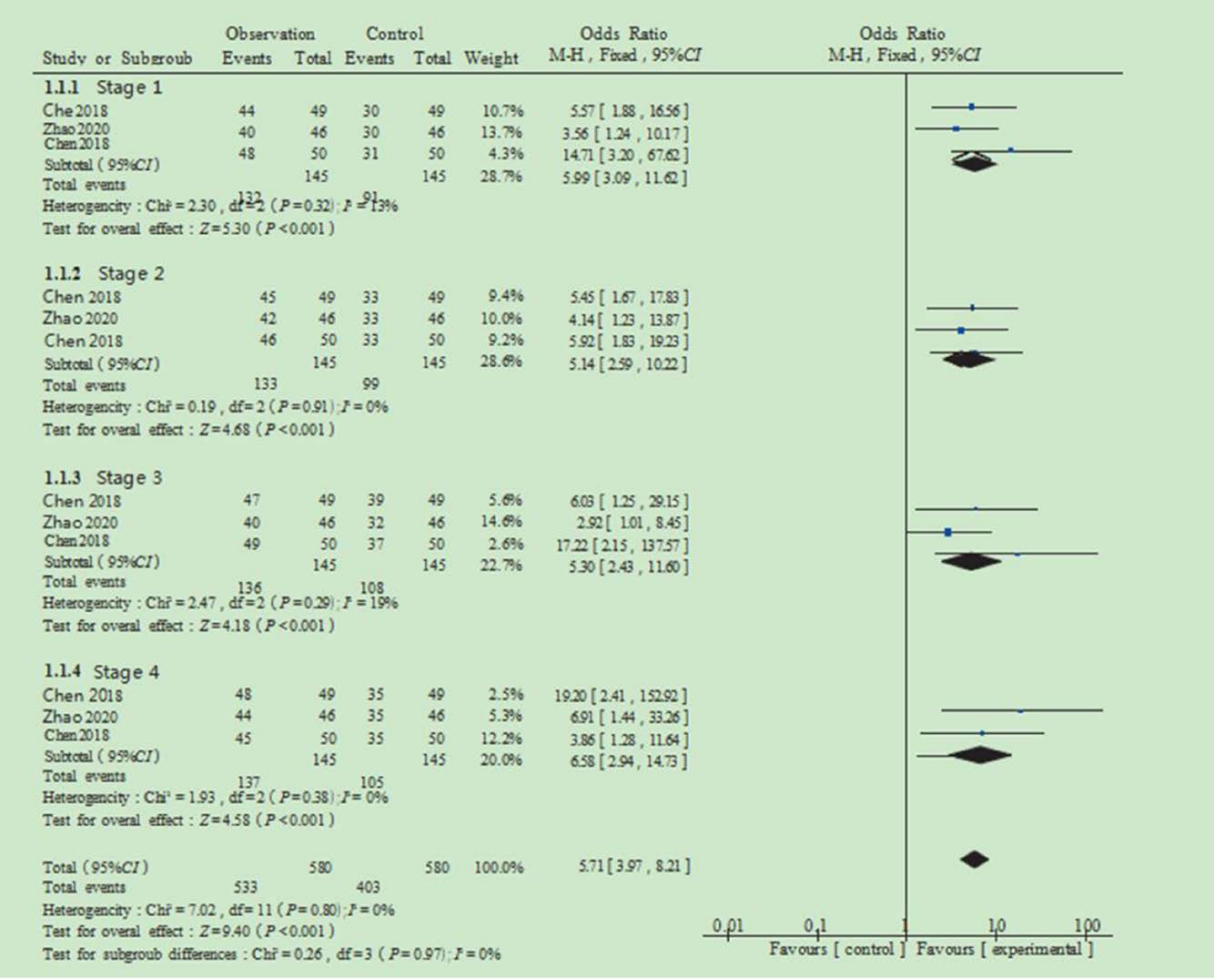
Forest plot of the impact of health education on the health knowledge of assisted reproduction.

Health knowledge awareness among patients was assessed across 4 stages: pre-pregnancy preparation, embryo transfer preparation, the embryo transfer procedure, and post-transfer care. Among the 11 studies included, 3 reported outcomes related to patient health knowledge. In stage 1 (pre-pregnancy preparation), 145 patients were included across the control and observation groups, with a combined OR of 5.99 and a 95% CI of 3.09 to 11.62. The heterogeneity test showed *I*^2^ = 13% and *P* = .32, suggesting low heterogeneity; thus, a FEM analysis was applied, yielding Z = 5.30 (*P* <.001), indicating a statistically significant difference. In stage 2 (embryo transfer preparation), the combined OR was 5.14, with a 95% CI of 2.59 to 10.22. The heterogeneity test showed *I*^2^ = 0% and *P* = .91, indicating no significant heterogeneity. FEM analysis yielded Z = 4.68 (*P* <.001), confirming statistical significance. In stage 3 (embryo transfer procedure), the pooled OR was 5.30, with a 95% CI of 2.43 to 11.60. The heterogeneity test showed *I*^2^ = 19% and *P* = .29, suggesting low heterogeneity. FEM analysis resulted in Z = 4.18 (*P* <.001), demonstrating statistical significance. In stage 4 (post-transfer care), the combined OR was 6.58, with a 95% CI of 2.94 to 14.73. The heterogeneity test showed *I*^2^ = 0% and *P* = .38, indicating no significant heterogeneity. FEM analysis yielded Z = 4.58 (*P* <.001), indicating statistical significance. The sub-combination of the 4 stages produced a Z score of 9.40 (*P* <.001), indicating a statistically significant difference in health knowledge awareness rates between the control and observation groups (Fig. 4).

### 3.5. Satisfaction

**Figure 5.**
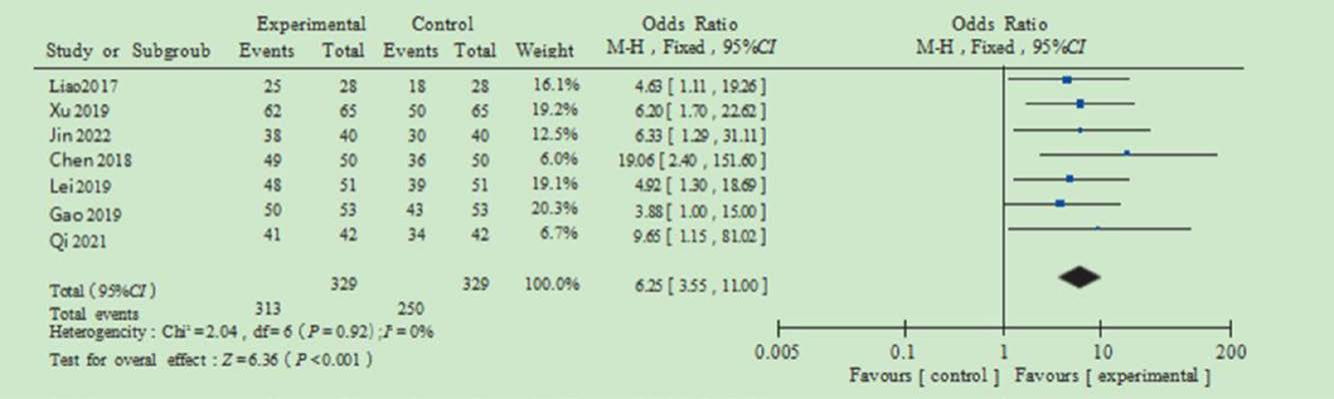
Forest plot of the impact of health education on assisted reproduction.

The pooled analysis of patient satisfaction following the intervention included data from 7 of the 11 studies, encompassing 329 patients in both the control and observation groups. The combined OR was 6.25, with a 95% CI of 3.55 to 11.00. The heterogeneity test showed *I*^2^ = 0% and *P* = .92, suggesting no significant heterogeneity among the included studies. Therefore, a FEM was applied, supporting statistically significant difference (Z = 6.36, *P* <.001) in satisfaction between the 2 groups (Fig. 5).

### 3.6. Evaluation of publication bias

**Figure 6.**
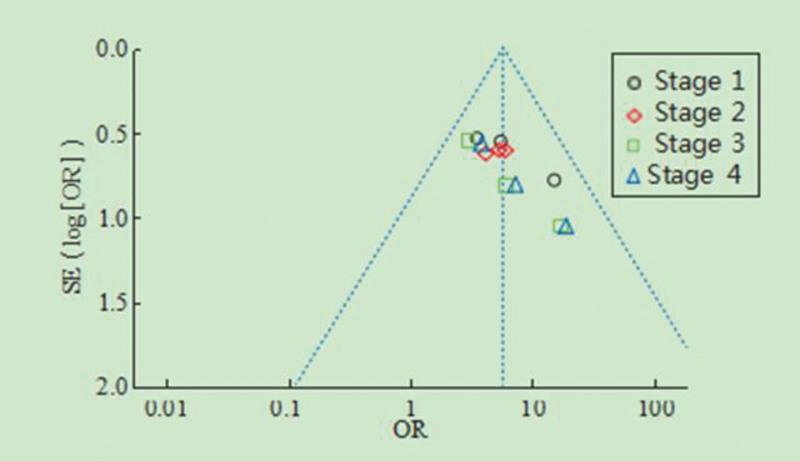
Funnel diagram of the effect of health education on the health knowledge of assisted reproduction.

The publication bias funnel chart of the combined results of the health knowledge awareness rate following the interventions is shown in Figure 6. The scatter points representing the included studies fall within the CI and are symmetrically distributed, indicating no apparent publication bias.

## 4. Discussion

ART includes artificial insemination, in vitro fertilization-embryo transfer (IVF-ET), and its derivatives.^[48]^ Clinically, patients with tubal infertility, unexplained infertility, endometriosis, male factor infertility, abnormal ovulation, and cervical factors^[39,40]^ are scheduled for IVF-ET when conventional treatments fail to achieve pregnancy.^[41,49]^ However, many patients receiving ART face substantial familial and societal pressure, leading to psychological distress and emotional instability.^[42,43,50]^ In addition, a general lack of knowledge about assisted reproduction often results in inadequate adherence to necessary precautions, potentially diminishing the success rate of ART procedures.^[44,47]^ This meta-analysis included 11 studies involving 1324 participants evaluating patient health cognition scores across 4 stages of ART: pre-pregnancy preparation, embryo transfer preparation, embryo transfer procedure, and post-transfer, and provides evidence that structured health education significantly improves cognitive understanding, health knowledge awareness, and patient satisfaction among infertile patients undergoing ART. The pooled analysis demonstrated substantial effect sizes across all measured outcomes, with health cognition scores showing particularly strong improvements (Z = 19.17, *P* <.001) across the 4 treatment stages.

Due to the use of different questionnaires across studies, SMD was employed as the effect size. SMD, defined as the ratio of the mean difference to the combined standard deviation, is unitless, which facilitates direct comparison of effect sizes across studies. This approach is particularly appropriate when outcome measures vary in scale or units, as it mitigates the influence of absolute values and enhances comparability. At stage 4 (post-transfer care), the pooled analysis yielded an SMD of 4.40, with a 95% CI of 3.94 to 4.86. The REM analysis showed a Z score of 18.89 (*P* <.001). The combined analysis across all 4 stages produced Z = 19.17 (*P* <.001), indicating a statistically significant difference in health cognition scores between the control and observation groups. These findings suggest that patients who received structured health education demonstrated significantly higher cognition scores than those in the control group at various stages of ART. Accordingly, incorporating tailored health education into nursing practice at each stage of ART may enhance patients’ understanding and engagement with the treatment process.^[45,46]^ In addition, the combined analysis of health knowledge awareness across the stages of pre-pregnancy preparation, embryo transfer preparation, and the embryo transfer procedure yielded a Z score of 9.40 (*P* <.001), indicating statistical significance between groups. Most patients undergo ART for the first time and often have little knowledge of the pregnancy cycle. Given the complexity and uncertainty of ART treatment, patients can easily lose confidence.^[51,52]^ Delivering stage-specific health education, covering key concepts and necessary precautions, can facilitate patient cooperation with medical personnel and foster a deeper understanding of both the condition and treatment pathway, ultimately contributing to improved outcomes.^[53]^

Our findings align with the broader literature on psychosocial interventions in reproductive medicine. Previous systematic reviews have consistently reported positive effects of educational and psychological support interventions on both psychological well-being and pregnancy outcomes in infertile populations.^[1,37,38]^ The significant improvement in health knowledge awareness rates (Z = 9.40, *P* <.001) observed in our analysis corroborates earlier research suggesting that targeted educational interventions enhance patients’ understanding of treatment procedures and self-care requirements.^[22,23]^

The analysis of patient satisfaction revealed no significant heterogeneity among the included studies (*I*^2^ = 0% and *P* = .92), justifying the use of FEM analysis. The pooled results showed a statistically significant difference between groups (Z = 6.36, *P* <.001), which indicates patients in the observation group who received health education possess higher levels of nursing satisfaction, consistent with patient-centered care models that emphasize the importance of information provision and emotional support in healthcare delivery.^[29,30]^ Accordingly, the implementation of health education at various stages of assisted reproduction is conducive to improving patient satisfaction. It also facilitates a better understanding of the patient’s condition and the treatment process, thereby improving overall health cognition. Moreover, health education may contribute to alleviating psychological distress, enhancing emotional well-being, and fostering greater cooperation with medical staff, ultimately helping to prevent or reduce the incidence of adverse pregnancy outcomes.

The stage-specific nature of our intervention aligns with the theoretical framework of adult learning, which emphasizes the importance of timely, relevant information delivery corresponding to patients’ immediate needs and concerns.^[31,32]^ The consistent positive effects across all 4 stages (pre-pregnancy preparation, embryo transfer preparation, embryo transfer procedure, and post-transfer care) suggest that comprehensive, phased educational approaches may be more effective than single-intervention models.

This review has several limitations. First, most assessed trials were from China, which may limit the generalizability of our findings to other populations and healthcare contexts. Cultural and system-specific factors can influence patient engagement, communication with healthcare providers, and response to educational interventions, potentially affecting external applicability. Second, considerable variability existed in the measurement instruments used, with some studies employing locally developed tools without established validity. Although the SMD was used to address differences in scale, variations in psychometric properties and cultural adaptations may have influenced the precision of pooled estimates. Third, intervention duration and intensity varied substantially, from brief single sessions to multi-session programs, and most studies had short follow-up, preventing evaluation of long-term effects on psychological well-being or reproductive outcomes. Fourth, the included studies were generally small (60–200 participants), limiting statistical power and increasing uncertainty in effect estimates. Lastly, although risk-of-bias assessment indicated overall good methodological quality, several studies had unclear risk in allocation concealment and blinding of outcome assessors, which may have led to overestimation of effects.

Although the search strategy covered 6 major databases, it was limited to English and Chinese publications, potentially excluding relevant studies in other languages. Only randomized controlled trials were included to strengthen causal inference; however, this excluded quasi-experimental and observational studies that may better reflect real-world clinical practice, potentially affecting observed effects. The small number of included studies (n = 11) limits the statistical power to detect publication bias, and the funnel plot, assessed for health knowledge awareness rate, showed symmetry but does not exclude bias in other outcomes. Lastly, the absence of individual patient data precluded subgroup analyses by age, infertility duration, prior ART cycles, or fertility diagnosis, factors known to influence psychological responses and treatment outcomes.

Future research could focus on validating these findings in large, multi-center international trials that use standardized, culturally adapted measures and extended follow-up to assess lasting effects on psychological well-being and ART outcomes. Work is also needed to refine the content, delivery format, and timing of health education, comparing approaches such as technology-assisted delivery, peer support, and individualized versus group-based models to identify the most effective and practical strategies. In addition, implementation studies could explore how best to integrate these interventions into routine clinical practice, addressing organizational readiness, resource requirements, and sustainability to ensure long-term impact.

## 5. Conclusion

This systematic review and meta-analysis indicate that structured health education significantly improves health cognition, knowledge awareness, and patient satisfaction among individuals undergoing ART. The consistent benefits observed across different treatment stages suggest that delivering targeted education throughout the ART process can enhance patient understanding, cooperation, and overall treatment experience. While these findings support the integration of structured health education into routine ART care, the predominance of single-country, small-sample studies underscores the need for broader, higher-quality research to confirm generalizability and long-term effectiveness.

## Author contributions

**Data curation:** Ruihua Xie, Fangfang Xie.

**Formal analysis:** Fangfang Xie.

**Methodology:** Ruihua Xie, Fangfang Xie.

**Resources:** Ruihua Xie.

**Software:** Fangfang Xie.

**Writing – original draft:** Ruihua Xie, Fangfang Xie.
